# Thermal Reactivity of Neutral and Oxidized Ferrocenyl-Substituted Enediynes

**DOI:** 10.3390/molecules191118399

**Published:** 2014-11-12

**Authors:** Mehmet Emin Cinar, Guido Morbach, Michael Schmittel

**Affiliations:** Department Chemie-Biologie, Universität Siegen, Adolf-Reichwein-Str., Siegen D-57068, Germany

**Keywords:** enediyne, ferrocene, thermal electron transfer, monocation, DFT, differential scanning calorimetry (DSC)

## Abstract

The coupling of two equivalents of ethynylferrocene (**2**) with one equivalent of 1,2-diiodocyclohexene (**1**) and 1,2-diiodobenzene (**4**) using Sonogashira cross-coupling conditions led to 1,2-bis(ferrocenylethynyl)cyclohexene (**3**) and 1,2-bis(ferrocenylethy-nyl)benzene (**5**), respectively. At high temperatures enediynes **3** and **5** showed exothermic signals in differential scanning calorimetry (DSC) measurements, suggestive of intramolecular diradicaloid ring formation (Bergman (C^1^−C^6^) or Schreiner-Pascal (C^1^−C^5^) cyclizations). The oxidation of **3** and **5** to the mono-oxidized enediynes **3^+^** and **5^+^** decreased the onset temperatures drastically. Equally, 1-ferrocenylethynyl-2-(*p*-nitro-phenyl)ethynylbenzene (**8**) displayed a significant decrease in the onset temperature after oxidation to **8^+^**. Because the insoluble nature of the polymeric material formed in the thermolysis of the oxidized enediynes prevented characterization, the origin of this drastic effect was studied by DFT. Contrary to expectations, one-electron oxidation does not lower the barrier for intramolecular cyclization. Rather, the computations suggest that the polymerization is initiated by a bimolecular process.

## 1. Introduction

The discovery of the antibacterial and antitumor properties of the naturally occurring enediynes [1], such as calicheamicin, dynemicin and kedarcidin, has attracted the interest of many scientists over the last three decades [2,3,4,5,6,7,8,9,10,11,12,13,14,15,16]. Because of their ability to afford (under physiological conditions!) reactive benzenoid σ,σ-diradicals [17,18,19,20] that induce single and double strand DNA cleavage, a significant amount of interest has focused on the design of simple acyclic enediynes with similar properties. Notably, chelate complexation in acyclic enediynes bringing closer together the C^1^ and the C^6^ termini [21,22,23,24,25,26,27] lowered the cyclization temperature as did the use of smaller strained cyclic enediynes [28,29]. Moreover, ligand field effects were exploited to influence the reactivity of acyclic metalloenediynes [30]. While it has been widely documented that the temperature of the Bergman cyclization depends on the C^1^–C^6^ distance, the effect of varying electronic properties has not been fully understood yet [31,32,33,34,35,36,37]. Since the activation of neutral enediynes has been accomplished by photoinduced electron transfer (PET) [38], we evaluate herein the possibility to activate ferrocenyl enediynes by thermal one-electron oxidation, a methodology that has been successful in our hands over many years [39,40].

## 2. Results and Discussion

### 2.1. Synthesis of Enediynes **3**, **5** and **8**, and Their Oxidation to **3^+^**, **5^+^** and **8^+^**

**Figure 1:**
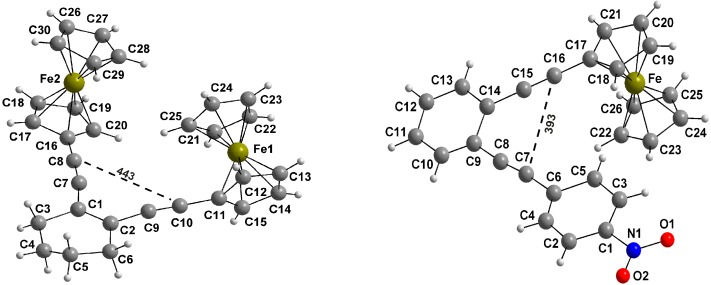
Crystal structures of **3** (**left**) and **8** (**right**). Critical distances are given in pm.

**Scheme 1:**
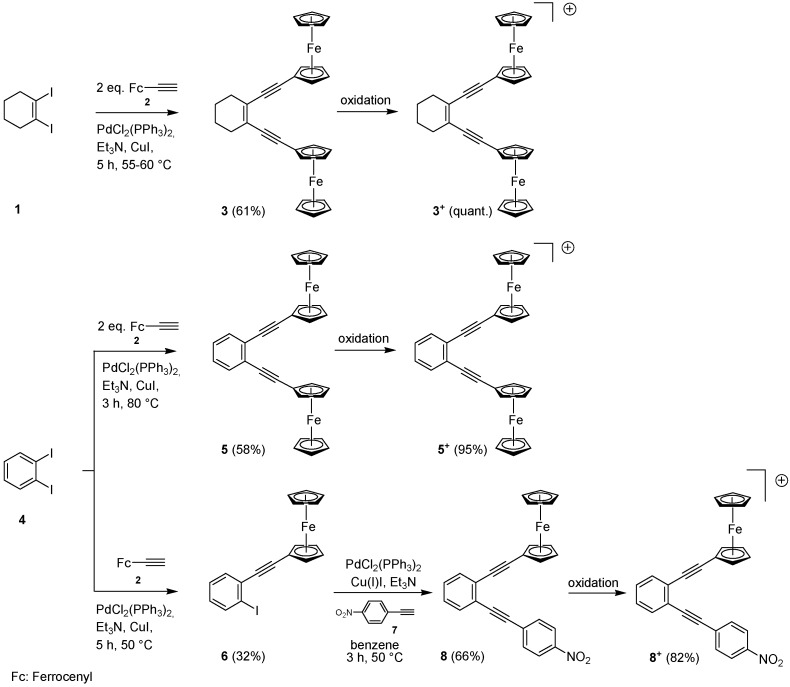
Synthesis of enediynes **3**, **5** and **8**, and their oxidation to **3^+^**, **5^+^** and **8^+^**.

The synthesis of enediynes **3** and **5** was accomplished by reacting two equivalents of ethynylferrocene (**2**) [41] in Sonogashira cross-couplings [42,43,44,45] with one equivalent of 1,2-diiodocyclohexene (**1**) or 1,2-diiodobenzene (**4**) [46], furnishing **3** and **5** in 61% and 58% yield, respectively. Two sequential Sonogashira couplings of 1,2-diiodobenzene (**4**) [47], first with ethynylferrocene (**2**) and then *p*-nitrophenylacetylene (**7**) [48,49] provided **8** (Scheme 1). The structures of **3** and **5** were unambiguously established by X-ray crystallography (Figure 1). Their oxidation using tris(4-bromophenyl)aminium hexachloroantimonate or tris(4-tolyl)aminium hexafluoroantimonate as one-electron oxidant furnished **3^+^**, **5^+^** and **8^+^** in high yield.

**Figure 2:**
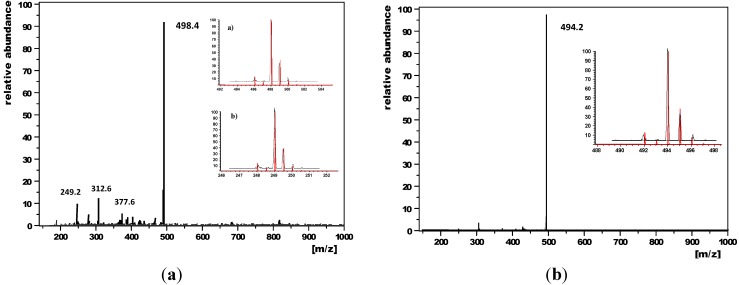
Electrospray ionization mass spectra of **3** (**left**) (insets displaying **3^+^** (**a**) and **3^2+^** (**b**)) and **5** (**right**) along with their isotopic distributions (red: calculated, black: experimental distribution).

Electrospray ionization mass spectra of **3**, **5** (Figure 2) and **8** (Figure S7) showed in all cases the signal of the mono-oxidized enediynes **3^+^** (498.4), **5^+^** (494.2) and **8^+^** (431.4). The spectrum of **3^+^** displayed additionally a very weak signal, which belongs to the doubly oxidized enediyne **3**^2+^ (249.2).

### 2.2. Electrochemical Properties of **3**, **5** and **8**

**Figure 3:**
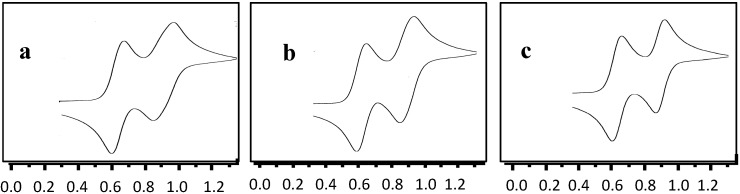
Cyclic voltammograms of (**a**) **3**; (**b**) **5** and (**c**) **8** in CH_3_CN at 100 mV·s^−1^. In each case, the cyclic voltammogram shows the wave of 1,1'-dimethylferrocene (lower potential) in presence of the enediyne (higher potential). (Potentials are given with respect to TPP = 2,4,6-triphenylpyrylium tetrafluoroborate).

Electrochemical investigations of compounds **3**, **5** and **8** in CH_3_CN were carried out using cyclic voltammetry at scan rates of 20, 50, 100, 200 and 500 mV·s^−1^. While the symmetrical enediynes exhibited reversible oxidation waves at *E*_1/2_^ox^ (**3**) = +0.90 V_TPP_ and *E*_1/2_^ox^ (**5**) = +0.89 V_TPP_, the push–pull system **8** displayed reversible oxidation and reduction waves at *E*_1/2_^ox^ (**8**) = +0.90 V_TPP_ and *E*_1/2_^red^ (**8**) = −0.63 V_TPP_, respectively. The amount of charge transfer in the oxidation was gauged against 1,1'-dimethylferrocene (1 equiv.) showing a ratio of current ratio of 0.98 for **3**, 0.96 for **5** and 1.0 for **8** (Figure 3). Because compound **8** contains only one ferrocenyl unit, but shows the same current ratio against the internal standard as **3** and **5**, the results clearly support a one-electron oxidation of **3**, **5** and **8** yielding the monocations **3^+^**, **5^+^** and **8^+^**. This finding is supported by the computational results demonstrating equal spin density at both ferrocene units in the cations, but contrasts other interpretations in the literature claiming no electronic communication between the ferrocene groups [50]. The latter studies, however, were done in CH_2_Cl_2_ as a strongly ion-pairing solvent.

### 2.3. Thermal Properties

**Figure 4:**
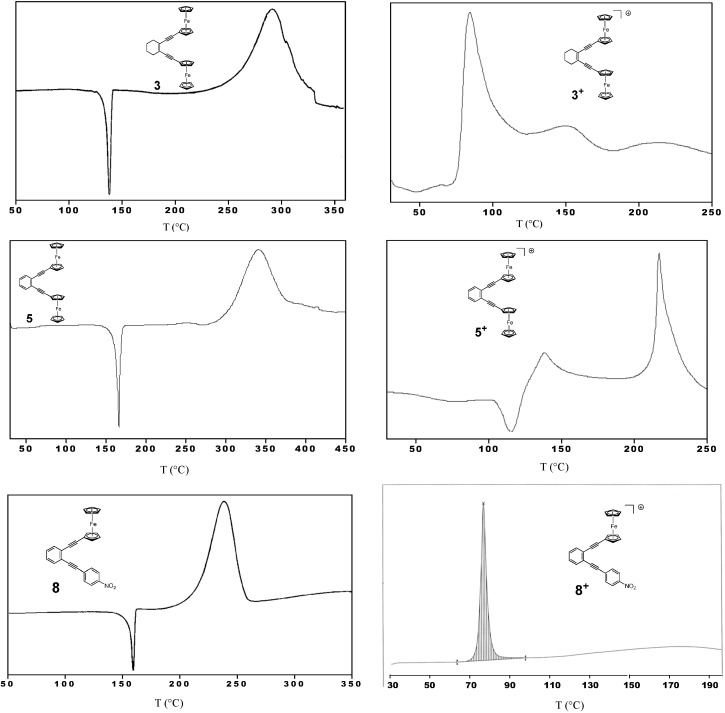
DSC graphs of **3**, **5** and **8** and their monocations **3^+^**, **5^+^** and **8^+^** (heating rate: 10 °C·min^−1^).

To evaluate the thermal stability of both the neutral and mono-oxidized enediynes we used differential scanning calorimetry (DSC) at a heating rate of 10 °C·min^−1^ (Figure 4). For the neutral enediynes **3**, **5** and **8** the *T_onset_* of the exothermic signals were determined to be 262, 300 and 216 °C. These onset temperatures are in good agreement with those of structurally related diaryl enediynes undergoing Bergman cyclization [35]. Equally, the trend is the same. With increasing electron density pushed into the enediyne by the substituents, the temperature of cyclization is raised. In contrast, the *T_onset_* temperatures of the oxidized enediynes **3^+^**, **5^+^** and **8^+^** were recorded at 77, 128 and 76 °C, respectively. Unfortunately, the analysis of the thermolyzed samples showed insoluble polymeric material that excluded any routine organic characterization.

### 2.4. Computations

To shed light on the different thermal stability of the neutral and mono-oxidized enediynes, we conducted DFT calculations on both **3** and **3^+^**. By comparing the most common DFT methods in regard to accuracy and computational costs for the Bergman and Myers-Saito reactions, Schreiner *et al.* [51] established the pure DFT functionals (MPWLYP, G96LYP, and BLYP) as the most suitable methods. Therefore, the BLYP method with a 6-31G(d,p) basis set combined with LANL2DZ was chosen for computing the stationary points in our study. Unless otherwise stated, full geometry optimization of all gas-phase stationary points was performed with 6-31G(d,p) and LANL2DZ basis sets using Becke’s pure gradient-corrected exchange functional [52] in conjunction with the Lee-Yang-Parr non-local correlation functional (BLYP) [53] as implemented in Gaussian 09 [54]. For all open-shell singlet state transition state (TS) structures, intermediates and cation radicals we used unrestricted calculations with a broken spin symmetry approach (BS-UBLYP) involving the mixing of the frontier molecular orbitals (HOMO and LUMO) to break the spin and spatial symmetries. The restricted method was applied for all closed-shell molecules. It is well known that the broken spin approach in combination with DFT provides good results for the cyclization of unsaturated systems [2,51,55,56,57]. The minima and transition states were verified by analyzing the harmonic vibrational frequencies, using analytical second derivatives, which have NIMAG = 0 and 1, respectively.

**Scheme 2:**
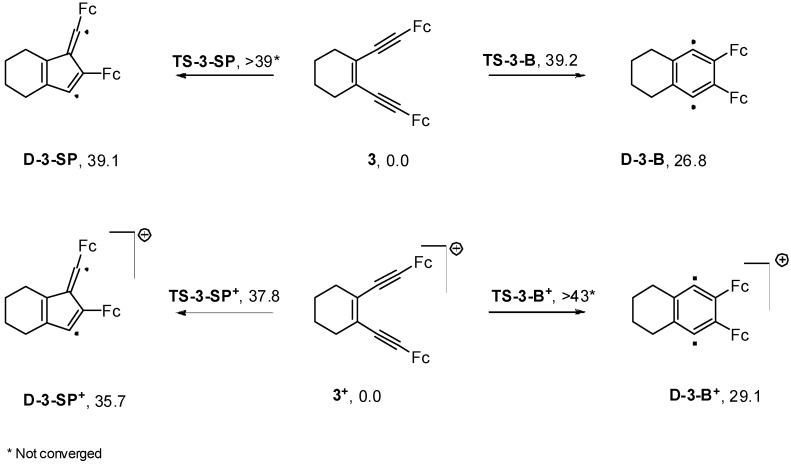
(U)BLYP/6-31G(d,p)/LANL2DZ computational results on the Bergman (**B**) and Schreiner-Pascal (**SP**) cyclizations of enediynes **3** and **3^+^** (electronic energies related to the starting enediynes are given in kcal·mol^−1^).

**Figure 5:**
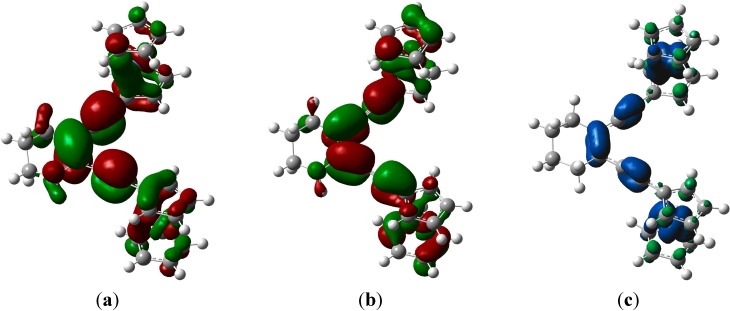
SOMO (**a**); LUMO (**b**) and spin density (**c**) of **3^+^**.

Based on the computational results, mono-oxidation of the enediyne **3** did not decrease the activation barrier for the Bergman or Pascal-Schreiner [51,58] cyclizations (Scheme 2). This phenomenon can be rationalized by the fact that a decrease of electron density occurs in the out-of-plane π-orbitals rather than diminishing electron repulsion between the in-plane π-orbitals (Figure 5). Interestingly, when we rotated the ferrocenyl group in **8^+^** to probe for an in-plane distonic cation radical, the spin density nevertheless remained in the out-of-plane orbitals. A minimum was located at 68° that was only 2.6 kcal·mol^−1^ higher in energy (Figure S9).

**Scheme 3:**
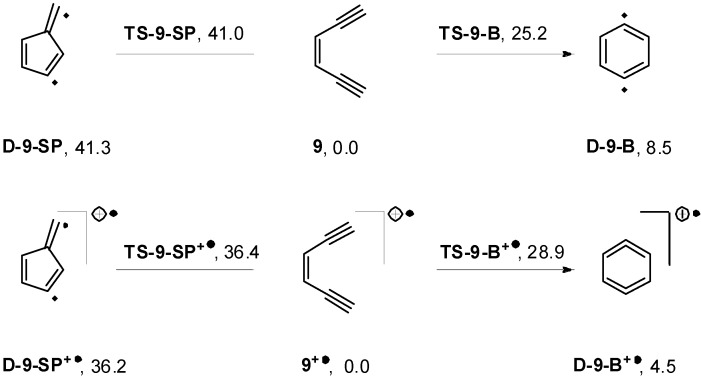
(U)BLYP/6-31+G(d) calculations on the Bergman (**B**) and Schreiner-Pascal (**SP**) cyclizations of cation radical **9^+•^** (Calculations on **9** at (UBS)-BLYP/6-31G(d) level from reference [56]. Relative energies with respect to enediynes are given in kcal·mol^−1^).

**Figure 6:**
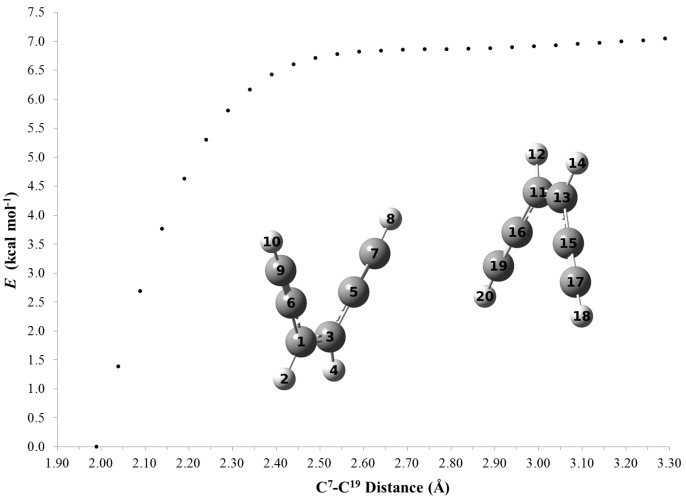
Analysis of the bond formation between C^7^ and C^19^ by performing the relaxed scan method at UBLYP/6-31+G(d) level on the reaction of **9^+•^** (**left**) and the neutral **9** (**right**) molecule (Relative energies are in kcal·mol^−1^ and distances are given in Å).

**Figure 7:**
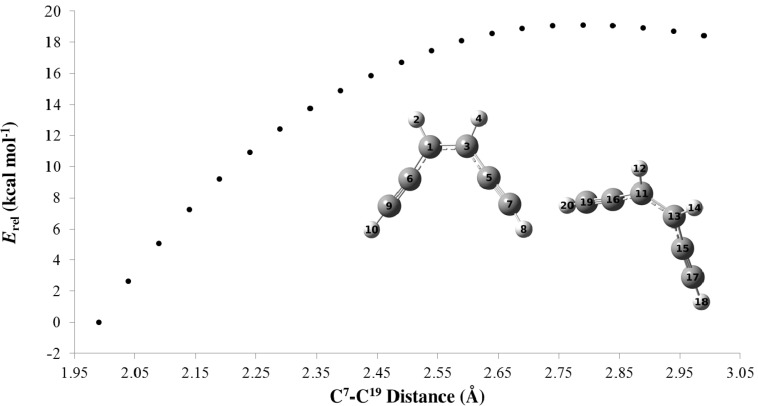
Analysis of the bond formation between C^7^ and C^19^ by performing the relaxed scan method at (UBS)-BLYP/6-31+G(d) level on the intermolecular reaction between two cation radicals **9^+•^** (Relative energies are in kcal·mol^−1^ and distances are given in Å).

To investigate the reason for the drastic change in the thermal reactivity of the monocations, we had a closer look on the parent enediyne **9**. The cation radical **9^+•^** exhibits an activation barrier of 28.9 kcal·mol^−1^ for the Bergman−analogous cyclization, which is higher than the TS of the neutral enediyne cyclization by 3.7 kcal·mol^−1^. In contrast, for the Schreiner-Pascal reaction, the cation radical exhibits a lower barrier (by 4.6 kcal·mol^−1^) than the neutral enediyne (Scheme 3). However, this rather small decrease cannot explain the low temperatures for cyclization as found experimentally. Hence, a plausible explanation for the experimental finding is to postulate an intermolecular dimerization initiating the polymerization. Notably, the DSC graphs display sharp exothermic signals in line with a chain reaction. Indeed, bringing neutral **9** and cation radical **9^+•^** molecules close together results in the corresponding product formation almost without any activation barrier (Figure 6). Alike two cation radicals **9^+•^** showed an almost barrierless (<0.7 kcal·mol^−1^) bond formation (Figure 7).

## 3. Experimental Section

### 3.1. General Information

Melting points were determined with a Büchi melting point apparatus and are uncorrected. ^1^H-NMR (200 MHz) and ^13^C-NMR spectra were recorded on a Bruker AC 200 instrument and chemical shifts are reported as δ values using the solvent CDCl_3_ (δ_H_ = 7.25/δ_C_ = 77.00) as internal reference. IR spectra were recorded on a Perkin Elmer 1750 FTIR. Column chromatography was carried out over silica gel (Merck, Darmstadt, Germany, SiO_2_, 0.063–0.200 mm, 70–230 mesh). TLC was performed using pre-coated silica gel SIL G/UV254 plates (Macherey-Nagel GmbH & Co. KG, Düren, Germany). ESI investigations were conducted on the LCQ Deca from Thermo Quest. Solvents for extraction and chromatography were technical grade and distilled prior to use. NEt_3_ was distilled over CaH_2_. All Sonogashira cross coupling reactions were carried out under dry nitrogen or argon in oven dried glassware. Cyclic voltammetry (CV) measurements were performed with a potentiostat (model 362, Fa. Princeton Applied Research) using a standard three electrode set-up (Pt working and Pt auxiliary electrode, silver wire as reference electrode). The experiments were carried out using a 1 mM solution of the enediyne in acetonitrile with 0.1 M tetra-*n*-butylammonium hexafluorophosphate as supporting electrolyte. All potentials were measured at a scan rate of 100 mV·s^−1^ and are referenced initially to triphenylpyrylium tetrafluoroborate (TPP, *E*_1/2_^red^ = −0.38 V_SCE_, *E*_1/2_^red^ = −0.75 V_Fc_) as internal standard. Ethynylferrocene (**2**) was synthesized according to the literature [41].

### 3.2. 1,2-Bis(ferrocenylethynyl)cyclohexene (**3**)

Under nitrogen, 1,2-diiodocyclohexene (**1**) [46] (397 mg, 1.19 mmol), bis(triphenylphosphine)palla-dium(II) chloride (167 mg, 2.38 µmol) and copper(I)iodide (90.7 mg, 476 µmol) were suspended in triethylamine (40 mL). Then, ethynylferrocene (**2**) [41] (500 mg, 2.38 mmol) was added and the mixture was stirred for 5 h at 55–60 °C. The solvent was evaporated under reduced pressure and the remaining residue was dissolved in dichloromethane (30 mL). The organic layer was washed three times with a 2.5% potassium cyanide solution (25 mL) and three times with water (25 mL). The combined organic layers were dried over anhydrous magnesium sulfate and concentrated under atmospheric pressure. The crude product was purified by column chromatography over silica gel with *n*-hexane/dichloromethane (4:1, *R*_f_ = 0.18) as eluent furnishing an orange solid in 61% yield (359 mg, 721 µmol) with a melting point of 131 °C (DSC). IR (KBr): ν = 3097 (m), 2930 (s), 2858 (m), 2830 (m), 2134 (s, C≡C), 1636 (m), 1474 (m), 1433 (m), 1410 (m), 1351 (m), 1261 (w), 1202 (w), 1176 (w), 1135 (w), 1106 (s), 1024 (s), 1001 (s), 931 (w), 915 (m), 817 (s), 476 (s) cm^−1^. ^1^H-NMR (CDCl_3_): δ = 1.68 (m, 4H), 2.33 (m, 4H), 4.22 (t, *J* = 2.0 Hz, 4H), 4.23 (s, 10H), 4.49 (t, *J* = 2.0 Hz, 4H). ^13^C-NMR (CDCl_3_): δ = 22.0, 30.4, 65.8, 68.7, 70.1, 71.4, 87.0, 92.0, 125.6. C_30_H_26_Fe_2_ (498.2): Calcd. C 72.32, H 5.26; found C 71.81, H 5.27.

### 3.3. Oxidation of 1,2-Bis(ferrocenylethynyl)cyclohexene **3** to the Monocation **3^+^**

1,2-Bis(ferrocenylethynyl)cyclohexene (**3**, 30.0 mg, 60.2 µmol) was dissolved in dry chloroform (3 mL) under nitrogen. Thereafter, tris(4-bromophenyl)aminium hexachloroantimonate (99.1 mg, 121 μmol) was added until the reaction mixture remained blue. The mixture was stirred for another 30 min resulting in the dark green solution. After the addition of dry *n*-hexane (5 mL), a precipitate formed that was filtered under nitrogen and washed with dry *n*-hexane (50 mL). Drying under vacuum afforded 50.0 mg (60.0 µmol, quantitative yield) of an olive green solid (C_30_H_26_Fe_2_SbCl_6_). IR (KBr): ν = 3106 (m, C_Fc_-H), 2931 (m), 2859 (w), 2201 (m, C≡C), 1467 (s), 1418 (m, C=C), 1397 (w), 1352 (w), 1263 (w), 1181 (w), 1090 (w), 1030 (w), 1008 (w), 917 (w), 854 (s, ferrocene), 651 (w) cm^−1^. ESI (C_30_H_26_Fe_2_^+^): Calcd. 498.2; found 498.1.

### 3.4. 1,2-Bis(ferrocenylethynyl)benzene (**5**)

Under nitrogen, 1,2-diiodobenzene (**4**, 148 mg, 449 µmol), bis(triphenylphosphine)palladium(II) chloride (15.8 mg, 22.5 µmol) and copper(I)iodide (9.00 mg, 47.3 µmol) were suspended in triethylamine (5 mL) at ambient temperature. Then, ethynylferrocene (**2**) [41] (200 mg, 952 μmol) in anhydrous triethylamine (8 mL) was added to the yellow suspension. The reaction mixture was stirred at 80 °C for 3 h. After removal of the solvent, the remaining residue was dissolved in dichloromethane. The organic layer was washed three times with a 2.5% potassium cyanide solution (30 mL) and three times with water (30 mL). The combined organic layers were dried over anhydrous magnesium sulfate and concentrated under atmospheric pressure. The crude product was purified by column chromatography over silica gel with *n*-hexane/dichloromethane (4:1, *R*_f_ = 0.1) as eluent furnishing an orange solid in 58% yield (128 mg, 259 µmol). M.p.: 166 °C (DSC). IR (KBr): ν = 3037 (m, arom. =C-H), 2202 (m, C≡C), 1555 (m, C=C), 1478 (m, C=C), 1431 (s, ferrocene), 1002 (s, ferrocene), 814 (m, ferrocene) cm^−1^. ^1^H-NMR (CDCl_3_): δ = 4.25 (s, 10H), 4.27 (t, *J* = 1.8 Hz, 4H), 4.57 (t, *J* = 1.8 Hz, 4H), 7.25 (dd, *J* = 5.9, 3.4 Hz, 2H), 7.50 (dd, *J* = 5.9, 3.4 Hz, 2H). ^13^C-NMR (CDCl_3_): δ = 64.3, 67.9, 69.1, 70.5, 83.9, 91.3, 125.0, 126.3, 130.7. C_30_H_22_Fe_2_ (494.2): Calcd. C 72.91, H 4.49; found C 72.72, H 4.43.

### 3.5. Oxidation of 1,2-Bis(ferrocenylethynyl)benzene (**5**) to the Monocation **5^+^**

1,2-Bis(ferrocenylethynyl)benzene (**5**, 64.3 mg, 130 µmol) was dissolved in anhydrous chloroform (5 mL) under nitrogen atmosphere. Thereafter, tris(4-tolyl)aminiumhexafluoroantimonate (136 mg, 260 mol) was added until the reaction mixture remained blue. The mixture was stirred for another 30 min, while the solution turned to dark green. After the addition of anhydrous *n*-hexane (5 mL), a precipitate formed, which was filtered under nitrogen and washed with anhydrous *n*-hexane (50 mL). Drying under vacuum yielded 90.0 mg (123 µmol, 95%) of an olive green solid (C_30_H_22_Fe_2_SbF_6_). IR (KBr): ν = 3105 (s), 2210 (m, C≡C), 1601 (s), 1561 (m), 1476 (s), 1418 (m), 1322 (m), 1191 (m), 1070 (s), 1007 (s), 986 (m), 853 (s), 824 (m), 756 (s) cm^−1^. ESI (C_30_H_22_Fe_2_^+^): Calcd. 494.2; found 494.2.

### 3.6. 2-Ferrocenylethynyl-1-iodobenzene (**6**)

A mixture of ethynylferrocene (**2**) [41] (200 mg, 952 μmol), bis(triphenylphosphine)palladium(II) chloride (67.0 mg, 95.7 μmol), copper(I)iodide (36.3 mg, 191 μmol), and 1,2-diiodobenzene (**4**, 330 mg, 1.00 mmol, 131 μL) in anhydrous triethylamine (10 mL) was prepared under nitrogen and stirred at 50 °C for 5 h. After removal of the solvent under reduced pressure, 100 mL of dichloromethane was added. The organic phase was extracted with 2.5% aqueous KCN solution (3 × 70 mL) and washed with water (3 × 70 mL). The combined organic layers were dried over anhydrous magnesium sulfate, thereafter the solvent was evaporated under atmospheric pressure. The crude product was purified by column chromatography over silica gel with *n*-hexane/dichloromethane (4:1, *R*_f_ = 0.30) as eluent resulting in the title compound in 32% yield (127 mg, 308 μmol). IR (film): ν = 3104 (m, C-H), 2925 (m, C-H), 2223 (s, C≡C), 1628 (m), 1551 (w), 1483 (s), 1443 (s), 1429 (s), 1412 (s), 1293 (w), 1200 (w), 1171 (w), 1104 (s), 1016 (s), 1000 (s), 922 (s), 816 (s), 759 (s), 710 (w), 649 (w), 549 (m), 520 (s), 498 (s), 485 cm^−1^ (s). ^1^H-NMR (CDCl_3_): δ = 4.28 (t, *J* = 1.8 Hz, 2H), 4.32 (s, 5H), 4.60 (t, *J* = 1.8 Hz, 2H), 7.00 (td, *J* = 7.4, 1.5 Hz, 1H), 7.31 (td, *J* = 7.4, 1.0 Hz, 1H), 7.50 (dd, *J* = 7.4, 1.5 Hz, 1H), 7.88 (dd, *J* = 7.4, 1.0 Hz, 1H). ^13^C-NMR (CDCl_3_): δ = 64.5, 69.9, 70.0, 71.5, 87.7, 92.6, 100.1, 127.7, 128.7, 130.3, 132.2, 138.6. HRMS (C_18_H_13_FeI): Calcd.: 411.9412; found: 411.9410.

### 3.7. 2-(4-Nitrophenylethynyl)-1-ferrocenylethynylbenzene (**8**)

2-Ferrocenylethynyl-1-iodobenzene (**6**, 93.0 mg, 226 μmol), bis(triphenylphosphine)palladium(II) chloride (16.0 mg, 22.9 μmol) and copper(I)iodide (9.00 mg, 47.3 μmol) in anhydrous triethylamine (25 mL) were combined under nitrogen at ambient temperature. Then *p*-nitrophenylacetylene (**7**) (33.1 mg, 225 μmol) was added and the reaction temperature was increased up to 50 °C for 3 h. After removal of the solvent under reduced pressure, the residue was dissolved in dichloromethane. The organic phase was extracted with 2.5% aqueous KCN solution (3 × 70 mL) and washed with water (3 × 70 mL). The combined organic layers were dried over anhydrous magnesium sulfate and the solvent was evaporated under atmospheric pressure. The crude product was purified by column chromatography over silica gel with *n*-hexane/chloroform (1:1, *R*_f_ = 0.10) as eluent resulting in **8** in 66% yield (64.0 mg, 148 μmol) as a red solid with a melting point of 161 °C. IR (KBr): ν = 2975 (s), 2599 (s), 2496 (m), 2370 (m), 2202 (w, C≡C), 1590 (m), 1509 (s), 1481 (m), 1438 (m), 1397 (m), 1339 (s), 1172 (w), 1106 (s), 1034 (s) cm^−1^. ^1^H-NMR (CDCl_3_): δ = 4.20 (s, 5H), 4.29 (t, *J* = 1.8 Hz, 2H), 4.51 (t, *J* = 1.8 Hz, 2H), 7.30–7.36 (m, 2H), 7.53–7.58 (m, 2H), 7.74 (d, *J* = 8.9 Hz, 2H), 8.25 (d, *J* = 8.9 Hz, 2H). ^13^C-NMR (CDCl_3_): δ = 64.7, 69.2, 70.1, 71.5, 84.3, 91.1, 93.5, 94.0, 123.7, 124.1, 127.1, 127.4, 129.0, 130.4, 131.8, 132.0, 132.3, 147.7. HRMS (C_26_H_17_NO_2_Fe): Calcd. 431.0609; found: 431.0609.

### 3.8. Oxidation of 2-(4-Nitrophenylethynyl)-1-ferrocenylethynylbenzene **8** to the Monocation **8^+^**

2-(4-Nitrophenylethynyl)-1-ferrocenylethynylbenzene (**8**, 35 mg, 81.2 μmol) was dissolved in anhydrous chloroform (3 mL) under nitrogen. Then tris(4-bromophenyl)aminium hexachloroantimonate (66.3 mg, 81.2 μmol) was added until the reaction mixture remained blue. The reaction mixture was stirred for 1 h at room temperature. After the addition of anhydrous *n*-hexane (5 mL), a green precipitate formed that was filtered under nitrogen and washed with anhydrous *n*-hexane (50 mL) providing 50.9 mg (66.5 µmol, 82%) of an olive green solid (C_26_H_17_NO_2_FeSbCl_6_). IR (KBr): ν = 3102 (m), 2212 (m, C≡C), 1595 (m), 1515 (s), 1500 (m), 1420 (w, ferrocene), 1349 (s), 1286 (w), 1173 (m), 1108 (w), 1064 (w), 1002 (w), 853 (s), 761 (m), 749 (m), 688 (w) cm^−1^. ESI (C_26_H_17_NO_2_Fe^+^): Calcd. 431.3; found 431.4.

## 4. Conclusions

While neutral enediynes undergo thermal cyclization at high temperature in full agreement with the computed high activation barriers, the corresponding monocationic enediynes show thermal reactivity at much reduced temperatures. The DFT computations suggest, however, that monocations **3^+^**, **5^+^** and **8^+^** neither underwent a cation radical Bergman nor a cation radical Pascal-Schreiner cyclization. Rather they suggest that polymerization was initiated by an intermolecular bond formation, alike as computed for **9**^+•^.

## Supplementary Materials

Supplementary materials can be accessed at: http://www.mdpi.com/1420-3049/19/11/18399/s1.

## Supplementary Files

Supplementary File 1
