# Supplementary material for: Thermal Reactivity of Neutral and Oxidized Ferrocenyl-Substituted Enediynes
Source: Molecules. 2014 Nov 12;19(11):18399–413. doi: 10.3390/molecules191118399 (PMC6271431; doi:10.3390/molecules191118399)
Supplement: Supplementary File 1 [file molecules-19-18399-s001.pdf]

Supplementary Materials

NMR and ESI Spectra

Figure S1. ^{1}H-NMR (200 MHz) of compound **3**.

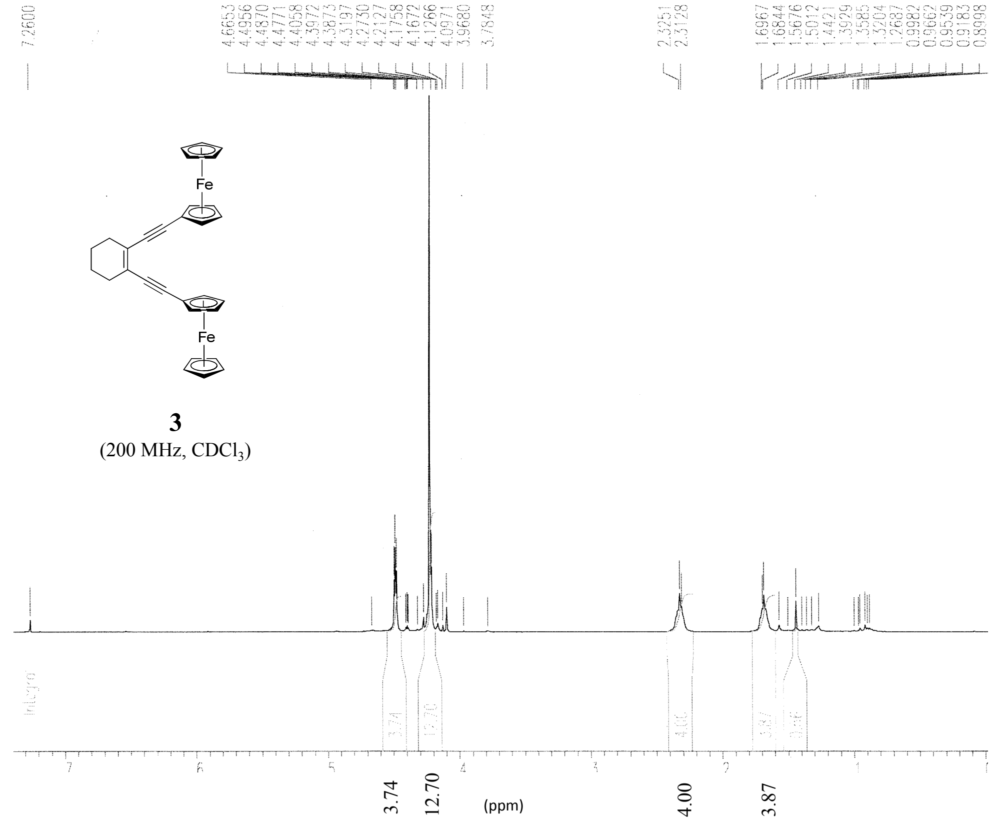

Figure S2. ^{13}C-NMR (50 MHz) of compound **3**.

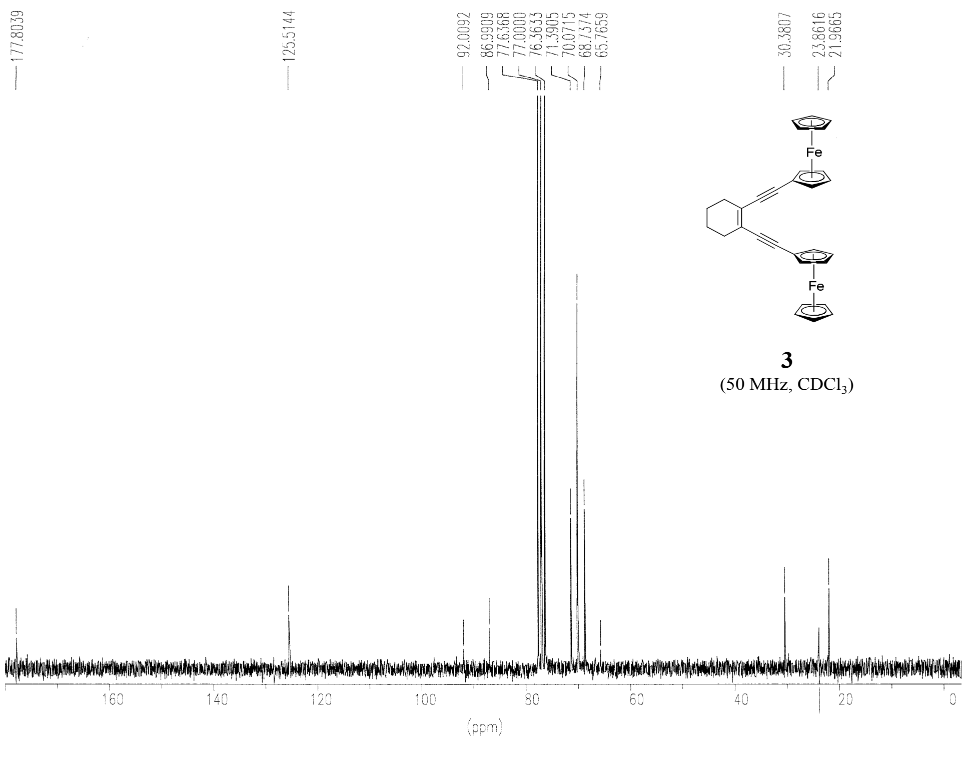

Figure S3. ^{1}H-NMR (200 MHz) of compound **5**.

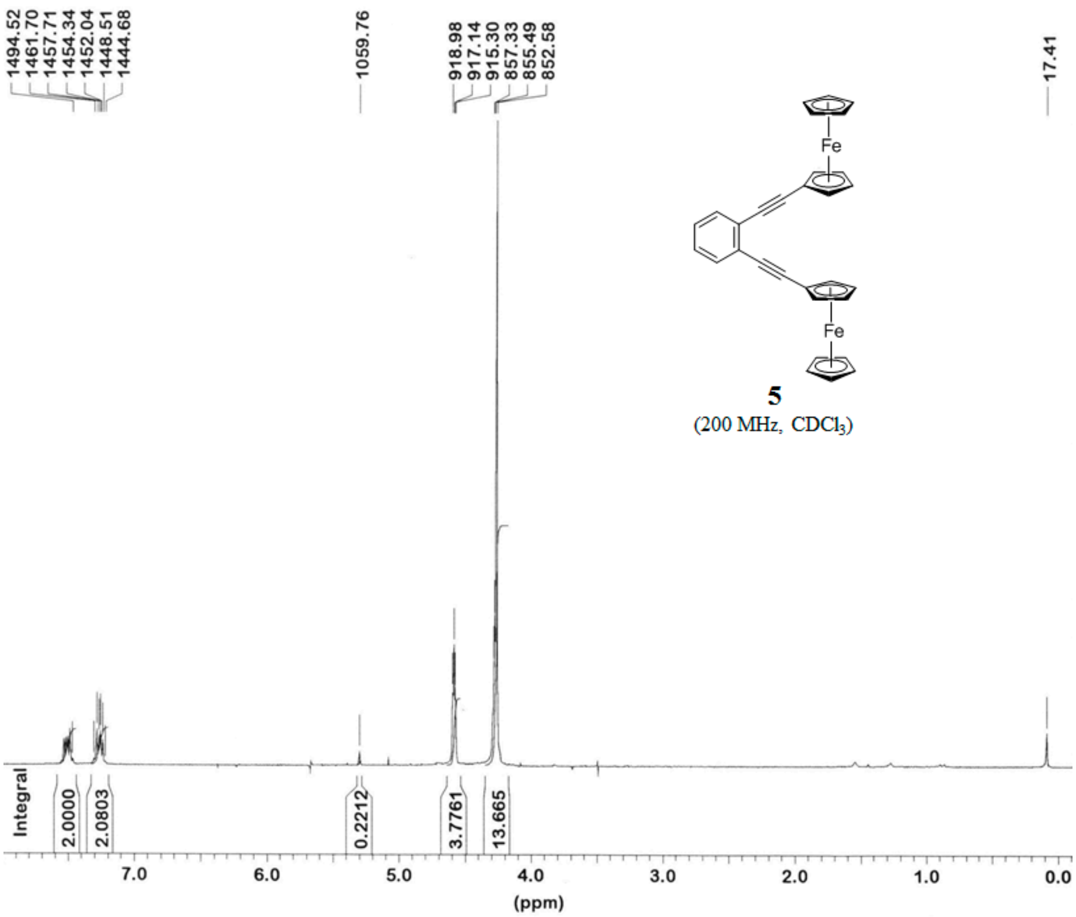

Figure S4. ^{1}H-NMR (200 MHz) of compound **6**.

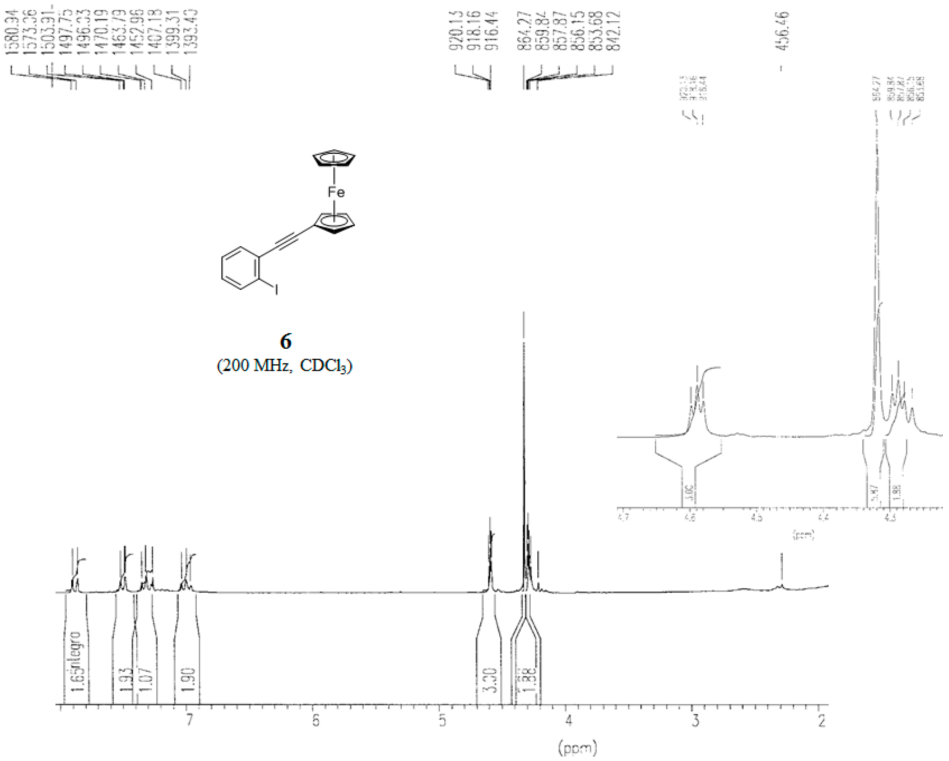

Figure S5. ^{1}H-NMR (200 MHz) of compound **8**.

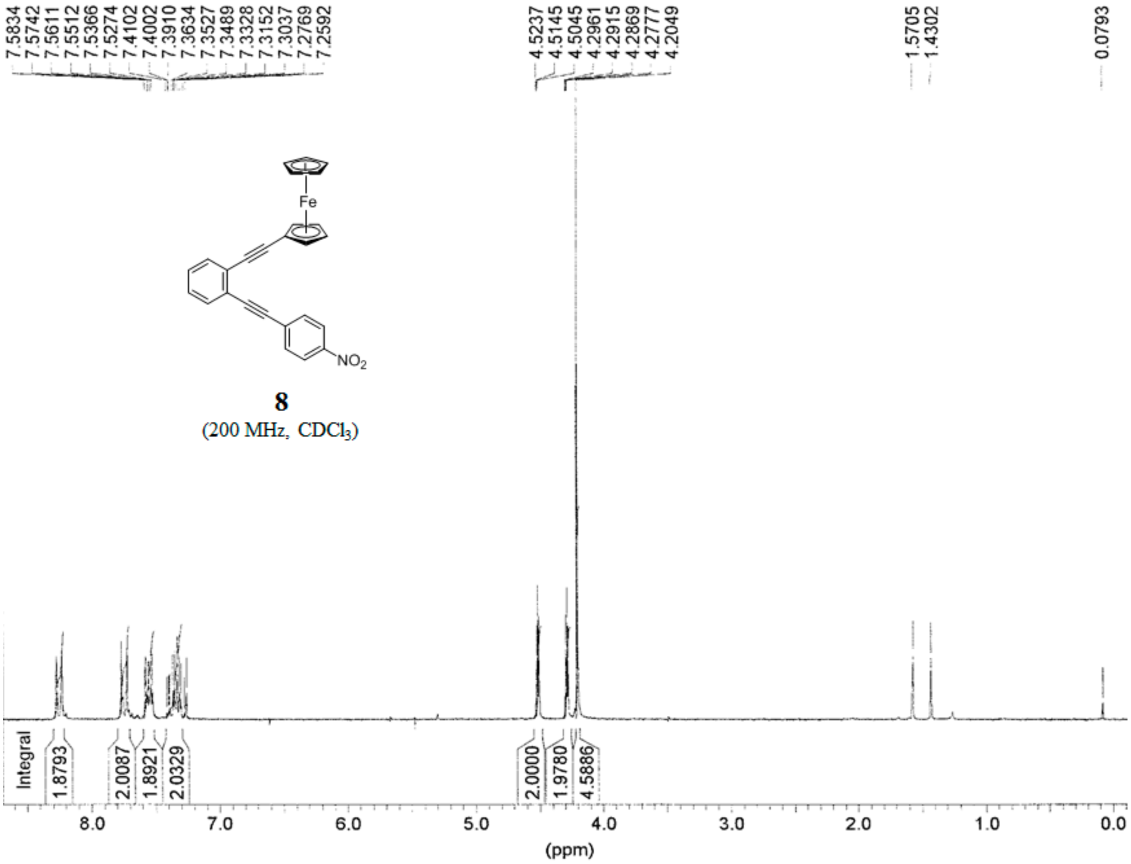

Figure S6. ^{13}C-NMR (50 MHz) of compound **8**.

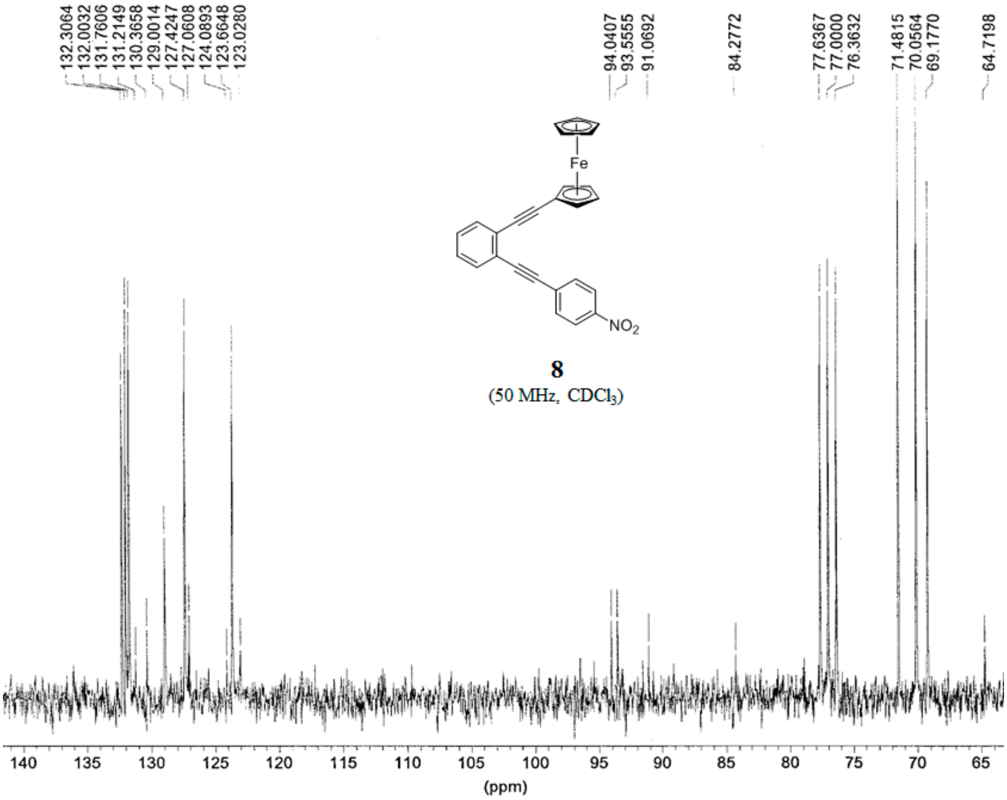

Figure S7. ESI-MS of compound **8**.

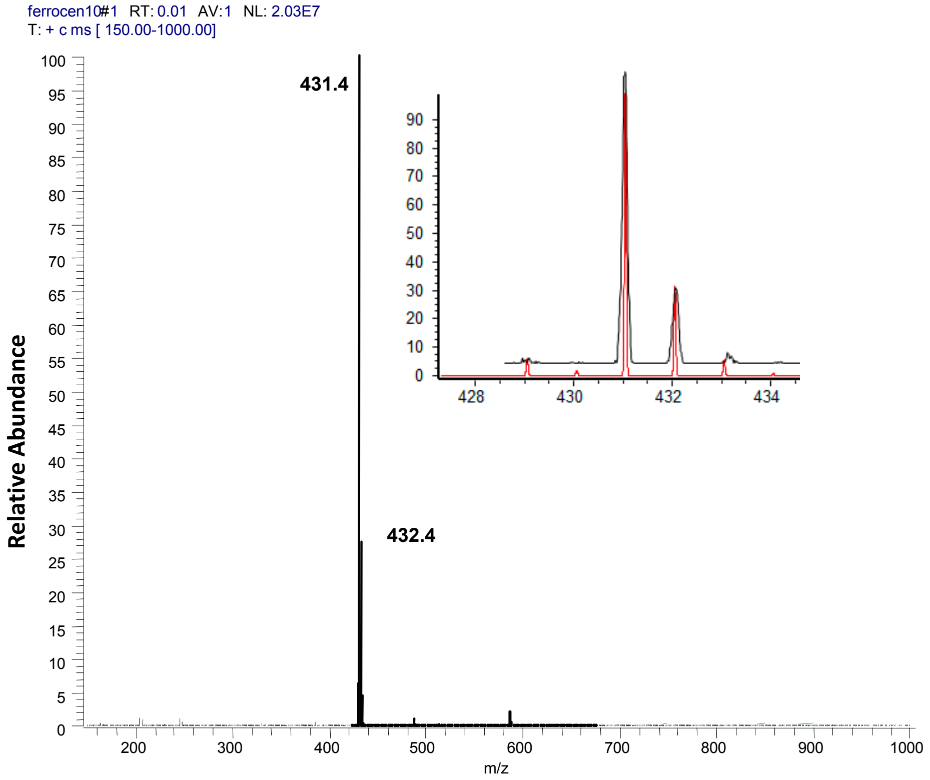

Table S1. X-Ray Data for **3**.

**Compound Name**	**1,2-Bis(ferrocenylethynyl)cyclohexene (3)**
Empirical formula	$C_{30}H_{26}Fe_2$
Formula weight/g·mol^{-1}	498.21
Temperature/K	170(1)
Wavelength/pm	71.073
Crystal system, Space group	monoclinic, P2$_1$/*n*, *m*P232
Lattice constants/pm/	a = 1149.3(2)
	b = 1159.4(2), β = 90.05(3)
	c = 1659.4(3)
Volume/nm^3	2.2111(7)
Z, Density (calculated/g·cm^{-3}	4, 1.497
Absorption coefficient/mm^{-1}	1.328
F(000)	1032
crystal size/mm^3	1.0 × 0.2 × 0.2
range (Θ)/°	3.02–30.48
range (H)	$-16 \leq h \leq 16, -16 \leq k \leq 16, -23 \leq l \leq 23$
Reflections collected/unique/significant	25777/6651/5934
$R_{int.}$, R_σ	0.0656, 0.0452
Completeness to Θ = 25.98	98.5
Absorption correction	None
Refinement method	Full-matrix least-squares on F^2
Data/Restraints/Parameter	6651/0/292

Table S1. *Cont.*

**Compound Name**	**1,2-Bis(ferrocenylethynyl)cyclohexene (3)**
$S(F^2)$	3.242
Final R indices ($I > 2\ \sigma\ (I)$)	R1 = 0.2549, wR2 = 0.6073
R indices (all data)	R1 = 0.2585, wR2 = 0.6126
$\Delta\rho_{min.}$, $\Delta\rho_{max.}/10^{-6}e\cdot pm^{-3}$	−1.823, 12,712

Table S2. X-Ray Data for **8**

**Compound Name**	**1-Ferrocenylethynyl-2-(4-nitrophenylethynyl)-benzene (8)**
Empirical formula	$C_{26}H_{17}NO_2Fe$
Formula weight/g·mol^{-1}	431.26
Temperature/K	153(2)
Wavelength/pm	71.069
Crystal system, Space group	orthorhombic, P*na*2$_1$, *o*P200
Lattice constants/pm/°	a = 729.40(10) α = 90
	b = 2583.1(5) β = 90
	c = 1056.6(2) γ = 90
Volume/nm^3	1.9908(6)
Z, Density (calculated/g·cm^{-3}	4, 1.439
Absorption coefficient/mm^{-1}	0.780
F(000)	888
crystal size/mm^3	0.31 × 0.25 × 0.23
range (Θ)/°	2.90–27.98
range (H)	$-9 \leq h \leq 9$, $-34 \leq k \leq 34$, $-13 \leq l \leq 13$
Reflections collected/unique/significant	26368/4679/3766
$R_{int.}$, R_σ	0.0420, 0.0305
Refinement method	Full-matrix least-squares on F^2
Data/Restraints/Parameter	4679/259/269
$S(F^2)$	1.071
Final R indices ($I > 2\ \sigma\ (I)$)	R1 = 0.0704, wR2 = 0.1840
R indices (all data)	R1 = 0.0878, wR2 = 0.2041
$\Delta\rho_{min.}$, $\Delta\rho_{max.}/10^{-6}e\cdot pm^{-3}$	−0.522(1), 1.138(1)

Computational Results—Data

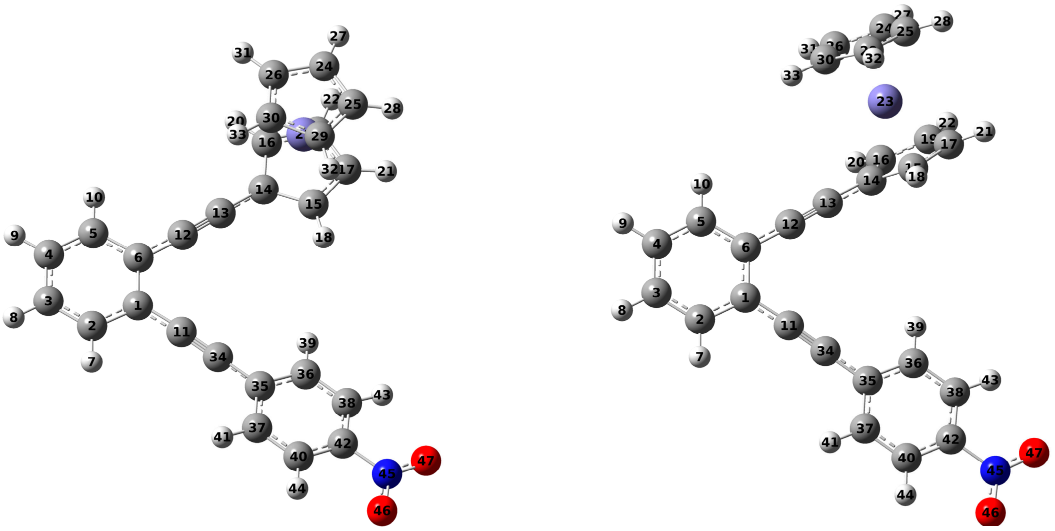

8$^+$ (<1-6-14-15 = −8°) **8**$^+$(II) (<1-6-14-15 = −68°)

Figure S8. Two rotamers of **8**$^+$.

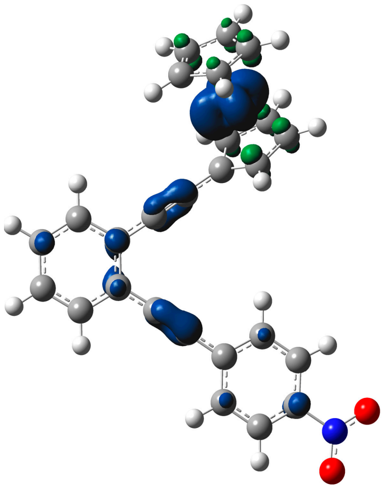

Figure S9. The plot of spin density of **8**$^+$(II) with a dihedral angle of 68°.

3

E: -1405.03301998 a.u.

C	-0.862504	1.980387	0.851020
C	0.862504	-1.980387	0.851020
C	-1.173247	2.707207	-0.324150
C	-0.766682	2.374842	-1.684221
C	-1.957794	3.933397	-0.408352
C	-1.323214	3.354275	-2.576095
C	-2.058102	4.313511	-1.790300
Fe	-0.062262	4.268617	-1.191048
C	0.828077	5.796935	-0.083784
C	0.727052	6.180984	-1.469994
C	1.629068	4.601913	-0.010350
C	1.463775	5.220889	-2.252217
C	2.019472	4.243389	-1.349633
C	1.173247	-2.707207	-0.324150
C	0.766682	-2.374842	-1.684221
C	1.957794	-3.933397	-0.408352
C	1.323214	-3.354275	-2.576095
C	2.058102	-4.313511	-1.790300
Fe	0.062262	-4.268617	-1.191048
C	-0.727052	-6.180984	-1.469994
C	-1.463775	-5.220889	-2.252217
C	-0.828077	-5.796935	-0.083784
C	-2.019472	-4.243389	-1.349633
C	-1.629068	-4.601913	-0.010350
C	-0.282454	0.633809	3.055742
C	0.282454	-0.633809	3.055742
C	-0.628498	1.368161	4.358584
C	0.628498	-1.368161	4.358584
C	0.062262	0.768599	5.603257
C	-0.062262	-0.768599	5.603257
C	-0.585936	1.317223	1.852028
C	0.585936	-1.317223	1.852028
H	-0.145613	1.524623	-1.957072
H	-2.394702	4.452935	0.441567
H	-1.187509	3.383443	-3.655589
H	-2.575876	5.190892	-2.173280
H	0.360873	6.306077	0.757050
H	0.170840	7.031916	-1.858921
H	1.852090	4.036197	0.892280
H	1.562810	5.220091	-3.336159
H	2.611052	3.374436	-1.631418
H	0.145613	-1.524623	-1.957072
H	2.394702	-4.452935	0.441567
H	1.187509	-3.383443	-3.655589
H	2.575876	-5.190892	-2.173280
H	-0.170840	-7.031916	-1.858921
H	-1.562810	-5.220091	-3.336159
H	-0.360873	-6.306077	0.757050
H	-2.611052	-3.374436	-1.631418
H	-1.852090	-4.036197	0.892280
H	-0.367261	2.434198	4.246509
H	-1.727668	1.344445	4.491917
H	1.727668	-1.344445	4.491917
H	0.367261	-2.434198	4.246509
H	-0.379151	1.199913	6.517532
H	1.131655	1.048195	5.603608
H	0.379151	-1.199913	6.517532
H	-1.131655	-1.048195	5.603608

TS-3-B

E: -1404.97063607 a.u.

C	0.902122	0.706459	-0.365899
C	-0.881052	0.693694	0.255028
C	1.487710	-0.618256	-0.550126
C	1.479239	-1.739954	0.371224
C	2.214733	-1.051627	-1.734322
C	2.158104	-2.845489	-0.248242
C	2.615065	-2.420178	-1.547063
Fe	3.438282	-1.211608	-0.063234
C	5.518786	-1.404253	-0.160219
C	5.041551	-1.792955	1.142831
C	5.132100	-0.033661	-0.385349
C	4.360932	-0.662178	1.723975
C	4.416202	0.422691	0.777723
C	-1.448945	-0.647175	0.376038
C	-1.543744	-1.662553	-0.656524
C	-2.013420	-1.222302	1.587423
C	-2.133057	-2.842451	-0.084276
C	-2.425602	-2.570682	1.300850
Fe	-3.438048	-1.224793	0.076276
C	-5.483500	-1.436671	0.457671
C	-5.202732	-1.723812	-0.926180
C	-5.059016	-0.085397	0.727270
C	-4.607025	-0.548866	-1.513218
C	-4.517281	0.461074	-0.490044
C	1.268646	1.947504	-0.550826
C	-1.258721	1.926782	0.465626
C	0.667009	3.177330	-0.308781
C	-0.666128	3.166494	0.247876
C	1.378390	4.500843	-0.617597
C	-1.383044	4.477608	0.593901
C	0.760956	5.727796	0.084933
C	-0.772158	5.725972	-0.075806
H	1.029209	-1.735587	1.360204
H	2.415507	-0.426117	-2.601234
H	2.329078	-3.821284	0.202387
H	3.186684	-3.020515	-2.252677
H	6.051588	-2.043033	-0.862288
H	5.151830	-2.776162	1.596565
H	5.323056	0.546155	-1.286327
H	3.867493	-0.640983	2.693706
H	3.936281	1.393621	0.887590
H	-1.218012	-1.538021	-1.686357
H	-2.106629	-0.700349	2.537383
H	-2.353737	-3.766595	-0.615025
H	-2.901567	-3.255129	2.000789
H	-5.913152	-2.128845	1.179579
H	-5.383779	-2.670722	-1.431508
H	-5.109272	0.422443	1.688411
H	-4.260616	-0.451187	-2.540227
H	-4.050173	1.439026	-0.593790
H	2.443060	4.399631	-0.351790
H	1.357920	4.655455	-1.713796
H	-1.363073	4.602788	1.693920
H	-2.447371	4.378915	0.325671
H	1.201137	6.650506	-0.329324
H	1.016353	5.708703	1.160250
H	-1.217049	6.635107	0.362706
H	-1.027568	5.733898	-1.151272

D3-B

E: -1404.99037173 a.u.

C	-0.660128	-1.366980	-4.361166
C	0.660163	1.366915	-4.361176
C	0.637987	0.434573	-5.593311
C	-0.637880	-0.434681	-5.593333
C	0.329952	0.648143	-3.048881
C	-0.330005	-0.648156	-3.048877
C	0.595075	1.186092	-1.806874
C	0.300649	0.668424	-0.552393
C	-0.300775	-0.668385	-0.552387
C	-0.595140	-1.186091	-1.806867
C	0.598262	1.442491	0.680381
C	-0.195527	1.554601	1.888136
C	1.777518	2.267519	0.873316
C	0.503968	2.402565	2.817946
C	1.723832	2.844201	2.190096
Fe	0.077055	3.447493	1.071751
C	0.118085	5.532710	0.961859
C	-1.091146	5.090402	1.609500
C	0.147797	4.962837	-0.361805
C	-1.810123	4.249474	0.684884
C	-1.044720	4.172492	-0.532781
C	-0.598327	-1.442468	0.680390
C	0.195514	-1.554579	1.888112
C	-1.777561	-2.267517	0.873364
C	-0.503931	-2.402564	2.817941
C	-1.723814	-2.844211	2.190136
Fe	-0.077072	-3.447466	1.071717
C	-0.118074	-5.532682	0.961804
C	1.091175	-5.090363	1.609401
C	-0.147849	-4.962795	-0.361852
C	1.810102	-4.249413	0.684765
C	1.044648	-4.172429	-0.532868
H	0.075781	-2.180041	-4.510978
H	-1.640648	-1.861656	-4.263088
H	-0.075730	2.179977	-4.511063
H	1.640682	1.861588	-4.263056
H	0.701314	1.038483	-6.514007
H	1.527334	-0.221961	-5.581790
H	-1.527228	0.221852	-5.581888
H	-0.701153	-1.038625	-6.514011
H	-1.158670	1.080653	2.057165
H	2.562812	2.409311	0.133810
H	0.154484	2.686228	3.808998
H	2.462137	3.515448	2.625074
H	0.887277	6.162331	1.405412
H	-1.394649	5.327249	2.627613
H	0.941363	5.088338	-1.095840
H	-2.752393	3.741655	0.881547
H	-1.293786	3.577381	-1.409184
H	1.158659	-1.080625	2.057107
H	-2.562883	-2.409313	0.133888
H	-0.154403	-2.686233	3.808977
H	-2.462091	-3.515474	2.625136
H	-0.887239	-6.162320	1.405381
H	1.394724	-5.327216	2.627499
H	-0.941443	-5.088298	-1.095856
H	2.752373	-3.741582	0.881396
H	1.293670	-3.577305	-1.409274

TS-3-SP

E: -1404.97017830 a.u.

C	-0.877945	0.342905	-0.328269
C	1.579915	1.280283	-0.801970
C	-1.525028	-0.886363	-0.392014
C	-2.247876	-1.441963	-1.542896
C	-1.718633	-1.839332	0.709314
C	-2.756936	-2.731652	-1.179046
C	-2.431636	-2.976398	0.203278
Fe	-3.592355	-1.255295	0.033477
C	-4.653206	-0.419664	1.639253
C	-5.321369	-1.604785	1.161040
C	-4.600129	0.526111	0.557026
C	-5.666134	-1.394049	-0.221003
C	-5.211533	-0.077727	-0.596003
C	2.275828	0.055716	-1.152563
C	1.863852	-1.330240	-1.035339
C	3.620853	0.040732	-1.709531
C	2.912158	-2.169270	-1.556215
C	3.998023	-1.320491	-1.975468
Fe	3.618897	-1.061461	0.052092
C	5.383412	-1.483973	1.088941
C	4.342201	-2.393723	1.491237
C	4.941014	-0.142913	1.378501
C	3.255373	-1.615001	2.030817
C	3.627751	-0.223872	1.964404
C	-0.304541	2.809394	-0.119106
C	0.886594	3.488168	-0.336901
C	-1.602614	3.472671	0.290991
C	1.020766	4.986028	-0.171574
C	-1.527072	5.009241	0.078888
C	-0.186766	5.588370	0.592110
C	-0.107773	1.376999	-0.363153
C	1.921239	2.560531	-0.738836
H	-2.323586	-0.961309	-2.515458
H	-1.330776	-1.708022	1.716815
H	-3.323253	-3.396241	-1.828896
H	-2.708659	-3.859197	0.776491
H	-4.252628	-0.270314	2.639863
H	-5.508623	-2.509299	1.736612
H	-4.128913	1.506017	0.590071
H	-6.158882	-2.111950	-0.873862
H	-5.307171	0.374660	-1.581050
H	0.926933	-1.683368	-0.618522
H	4.215233	0.930810	-1.900983
H	2.893611	-3.256889	-1.596545
H	4.948212	-1.651519	-2.390879
H	6.328068	-1.757417	0.622097
H	4.362194	-3.476712	1.382985
H	5.488784	0.773553	1.167535
H	2.311476	-2.007353	2.404693
H	3.004052	0.620716	2.250954
H	-2.448769	3.055720	-0.284393
H	-1.824780	3.254550	1.355355
H	1.111692	5.454397	-1.171536
H	1.966814	5.220032	0.347611
H	-2.377260	5.498459	0.583581
H	-1.626408	5.231002	-0.999307
H	-0.181333	6.687254	0.496877
H	-0.084864	5.363692	1.669643

D3-SP

E: -1404.97068762 a.u.

C	-0.908639	0.298163	-0.281333
C	1.500687	1.133097	-0.741251
C	-1.596278	-0.897271	-0.330975
C	-2.338323	-1.441330	-1.479779
C	-1.867956	-1.815134	0.788771
C	-2.890552	-2.710494	-1.106504
C	-2.601105	-2.941365	0.286060
Fe	-3.711375	-1.197935	0.063676
C	-4.809834	-0.350677	1.646616
C	-5.497669	-1.507715	1.126841
C	-4.694813	0.611452	0.584334
C	-5.787445	-1.265084	-0.261053
C	-5.278796	0.042068	-0.599926
C	2.168808	-0.123703	-1.060250
C	1.807707	-1.479141	-0.685671
C	3.400205	-0.221822	-1.827943
C	2.770389	-2.387504	-1.254348
C	3.754705	-1.608968	-1.961550
Fe	3.713478	-1.067715	0.044189
C	5.617373	-1.397761	0.835562
C	4.639978	-2.174343	1.555609
C	5.257333	-0.006279	0.955826
C	3.676200	-1.262539	2.120842
C	4.059548	0.076844	1.751436
C	-0.267349	2.738931	-0.126894
C	0.930905	3.389184	-0.378933
C	-1.551130	3.434690	0.267437
C	1.098321	4.887576	-0.268758
C	-1.448681	4.962835	0.005988
C	-0.092297	5.534764	0.485939
C	-0.060508	1.291120	-0.334460
C	1.933554	2.407070	-0.751225
H	-2.378224	-0.977308	-2.462470
H	-1.496934	-1.679887	1.802173
H	-3.462793	-3.369342	-1.757055
H	-2.914634	-3.805810	0.868496
H	-4.441881	-0.228437	2.663388
H	-5.732793	-2.414113	1.681499
H	-4.194528	1.575257	0.648860
H	-6.280433	-1.955363	-0.942860
H	-5.329335	0.514306	-1.579075
H	0.960943	-1.764829	-0.069439
H	3.938410	0.629319	-2.238476
H	2.768354	-3.470067	-1.140788
H	4.627004	-1.998092	-2.483729
H	6.463542	-1.793453	0.276583

H 4.619931 -3.259616 1.638386
H 5.779295 0.832295 0.499085
H 2.800942 -1.539247 2.705919
H 3.506287 0.987135 1.974096
H -2.407636 3.015838 -0.291453
H -1.773612 3.254530 1.338944
H 1.187274 5.321657 -1.284277
H 2.052618 5.122875 0.234981
H -2.283454 5.484469 0.503885
H -1.556330 5.151844 -1.077630
H -0.069907 6.629753 0.354937
H 0.017202 5.343214 1.569091

3$^+$

E: -1404.84080151 a.u.
C -0.943201 1.990312 0.758477
C 0.943150 -1.990354 0.758494
C -1.319013 2.758620 -0.359687
C -0.905990 2.554057 -1.742029
C -2.095461 3.993237 -0.330852
C -1.485227 3.593977 -2.543939
C -2.219200 4.477805 -1.675840
Fe -0.200770 4.433732 -1.116849
C 0.689844 5.986445 0.019744
C 0.523947 6.390508 -1.353700
C 1.551166 4.837593 0.041374
C 1.277915 5.481430 -2.177895
C 1.908191 4.516309 -1.312562
C 1.318961 -2.758630 -0.359693
C 0.905916 -2.554045 -1.742024
C 2.095437 -3.993231 -0.330894
C 1.485166 -3.593935 -2.543965
C 2.219169 -4.477766 -1.675895
Fe 0.200745 -4.433748 -1.116876
C -0.523937 -6.390529 -1.353787
C -1.277947 -5.481434 -2.177925
C -0.689808 -5.986527 0.019678
C -1.908218 -4.516361 -1.312535
C -1.551153 -4.837694 0.041378
C -0.315578 0.624934 2.928499
C 0.315602 -0.624935 2.928511
C -0.707889 1.329331 4.234336
C 0.707945 -1.329291 4.234361
C 0.016988 0.770713 5.478837
C -0.016897 -0.770632 5.478863
C -0.639574 1.294929 1.734480
C 0.639587 -1.294956 1.734506
H -0.282094 1.734958 -2.091833
H -2.527283 4.436805 0.563343
H -1.367554 3.708156 -3.619547
H -2.754871 5.374395 -1.980675
H 0.231776 6.459862 0.885799
H -0.076416 7.226892 -1.705845
H 1.831410 4.268650 0.925882
H 1.348871 5.510500 -3.263270
H 2.536364 3.686142 -1.628939
H 0.281999 -1.734951 -2.091802
H 2.527279 -4.436810 0.563285
H 1.367481 -3.708092 -3.619574
H 2.754855 -5.374338 -1.980757
H 0.076432 -7.226889 -1.705980
H -1.348933 -5.510462 -3.263298
H -0.231705 -6.459970 0.885702
H -2.536418 -3.686194 -1.628861
H -1.831379 -4.268789 0.925916
H -0.524360 2.410749 4.123882
H -1.802856 1.223687 4.357438
H 1.802915 -1.223646 4.357429
H 0.524410 -2.410712 4.123946
H -0.456362 1.174198 6.388006
H 1.065730 1.117376 5.484678
H 0.456480 -1.174086 6.388033
H -1.065638 -1.117294 5.484746

TS-3-B$^+$

E: -1404.77116571 a.u. (Not converged)
C 0.907718 0.635549 -0.601418
C -0.824107 0.591693 0.294480
C 1.557454 -0.635470 -0.818125
C 1.497128 -1.842199 -0.013501
C 2.530103 -0.882166 -1.872797
C 2.358317 -2.829038 -0.608735
C 2.994878 -2.234193 -1.758877
Fe 3.504407 -1.171393 -0.043349
C 5.529717 -1.561145 0.429981
C 4.706836 -1.702697 1.603973
C 5.394229 -0.201256 -0.040497
C 4.062513 -0.441672 1.854500
C 4.494254 0.480352 0.843830
C -1.430803 -0.722556 0.353081
C -1.592424 -1.653102 -0.752097
C -2.055464 -1.334982 1.529634
C -2.251028 -2.828442 -0.262209
C -2.523886 -2.636954 1.134707
Fe -3.494549 -1.159466 0.029698
C -5.518449 -1.610296 0.363224
C -5.267563 -1.456774 -1.051540
C -5.190779 -0.369201 1.017349
C -4.797240 -0.101360 -1.260971
C -4.751038 0.555284 0.015350
C 1.154616 1.886212 -0.871503
C -1.115703 1.826718 0.604934
C 0.614883 3.105316 -0.506976
C -0.594848 3.071909 0.292985
C 1.261531 4.441358 -0.890672
C -1.251988 4.368306 0.781026
C 0.808830 5.618605 -0.003451
C -0.728660 5.646684 0.091682
H 0.912053 -1.976069 0.891071
H 2.825827 -0.154180 -2.624457
H 2.515328 -3.840033 -0.238340
H 3.719075 -2.714863 -2.413399
H 6.128418 -2.340597 -0.037222
H 4.582727 -2.614382 2.184978
H 5.872003 0.210032 -0.927502
H 3.364515 -0.224324 2.660373
H 4.156576 1.508083 0.723870
H -1.266039 -1.472441 -1.773416
H -2.125567 -0.852894 2.502029
H -2.512649 -3.695163 -0.865417
H -3.036522 -3.339813 1.788079
H -5.865947 -2.524111 0.840353
H -5.404324 -2.201574 -1.832838
H -5.232890 -0.170487 2.086402
H -4.514121 0.326995 -2.220247
H -4.381700 1.563410 0.193962
H 2.356618 4.326772 -0.867443
H 1.001849 4.648761 -1.946358
H -1.088172 4.431590 1.873703
H -2.342867 4.278388 0.651218
H 1.200660 6.562677 -0.413522
H 1.239136 5.506782 1.008070
H -1.076509 6.530071 0.649959
H -1.157937 5.722803 -0.923178

D3-B$^+$

E: -1404.79444244 a.u.
C -0.704941 -1.338166 -4.345451
C 0.683760 1.360434 -4.339231
C 0.640456 0.431291 -5.572985
C -0.655791 -0.405950 -5.576514
C 0.335962 0.651348 -3.026797
C -0.359715 -0.633876 -3.029076
C 0.625185 1.166598 -1.783530
C 0.325891 0.650604 -0.530453
C -0.316219 -0.670984 -0.533279
C -0.631791 -1.169783 -1.790186
C 0.625555 1.426899 0.694371
C -0.169743 1.558188 1.895110
C 1.776730 2.294912 0.857447
C 0.512806 2.437569 2.805980
C 1.720511 2.891443 2.166025
Fe 0.066554 3.496035 1.054632
C 0.105826 5.589962 0.966814
C -1.115512 5.157056 1.597011
C 0.127434 5.052718 -0.371149
C -1.846388 4.359523 0.647392
C -1.082243 4.300874 -0.565293
C -0.624806 -1.442622 0.691919
C 0.164647 -1.569753 1.898557
C -1.777928 -2.306423 0.852750
C -0.524423 -2.445928 2.808437
C -1.727851 -2.901858 2.161161
Fe -0.057925 -3.502631 1.066240
C -0.087999 -5.598357 0.980243
C 1.122248 -5.152889 1.623759
C -0.097277 -5.067884 -0.359949
C 1.857953 -4.352437 0.679222
C 1.107051 -4.306367 -0.542856
H 0.013106 -2.167558 -4.487494
H -1.696854 -1.807928 -4.245062
H -0.037453 2.187145 -4.481752
H 1.673539 1.833737 -4.235230
H 0.715817 1.039694 -6.488147
H 1.516259 -0.242061 -5.565391
H -1.531293 0.267792 -5.571248
H -0.727541 -1.012170 -6.493456
H -1.131692 1.084318 2.072726
H 2.558449 2.435943 0.114290
H 0.162917 2.726310 3.795054
H 2.455205 3.573300 2.589453
H 0.878111 6.205902 1.422857
H -1.424880 5.380601 2.615965
H 0.921445 5.187400 -1.102767
H -2.798927 3.865060 0.827510
H -1.343492 3.733506 -1.456385
H 1.124648 -1.093816 2.080735
H -2.553957 -2.452413 0.104578
H -0.181765 -2.731577 3.800908
H -2.463790 -3.584819 2.580634
H -0.861661 -6.217423 1.429689
H 1.422041 -5.372472 2.646417
H -0.883991 -5.207803 -1.098455
H 2.805618 -3.851551 0.866844
H 1.372119 -3.738664 -1.432692

TS-3-SP$^+$

E: -1404.78058586 a.u.
C -0.998676 0.325631 -0.133244
C 1.548970 1.158531 -0.861910
C -1.657842 -0.899954 -0.152421
C -2.252729 -1.557926 -1.324645
C -1.983763 -1.740028 1.010715
C -2.820579 -2.799566 -0.893476
C -2.656266 -2.912048 0.533247
Fe -3.743374 -1.181155 0.070929
C -5.020339 -0.272350 1.475641
C -5.615874 -1.476856 0.955889
C -4.808992 0.624199 0.371524
C -5.759929 -1.324453 -0.468952
C -5.252190 -0.026550 -0.832464
C 2.144687 -0.094193 -1.181516
C 1.777502 -1.432792 -0.752080
C 3.423507 -0.224341 -1.880042
C 2.731466 -2.364124 -1.288839
C 3.741104 -1.619133 -1.993202
Fe 3.718127 -1.061658 0.019141
C 5.641335 -1.506829 0.760887
C 4.661563 -2.278392 1.479142
C 5.359372 -0.109597 0.986984
C 3.777534 -1.358282 2.148778
C 4.220795 -0.026483 1.856756
C -0.262876 2.776826 -0.045714
C 0.936251 3.399124 -0.439236
C -1.467056 3.538327 0.463680
C 1.136707 4.896796 -0.371751
C -1.350972 5.060742 0.182030
C 0.064702 5.590726 0.505370
C -0.219341 1.336173 -0.209986
C 1.874633 2.441188 -0.932395
H -2.210765 -1.175437 -2.341653

H -1.709525 -1.516437 2.039079
H -3.329266 -3.518032 -1.533019
H -3.019393 -3.729553 1.152704
H -4.774157 -0.077390 2.517299
H -5.887767 -2.357387 1.534474
H -4.354165 1.610695 0.432470
H -6.158233 -2.070253 -1.153851
H -5.210077 0.386241 -1.838170
H 0.908621 -1.688716 -0.153836
H 3.978970 0.610822 -2.298976
H 2.705238 -3.444133 -1.158908
H 4.611945 -2.034199 -2.496685
H 6.448200 -1.904960 0.148882
H 4.592134 -3.364015 1.502638
H 5.912099 0.731132 0.573279
H 2.919275 -1.627171 2.761126
H 3.734021 0.891965 2.180241
H -2.389543 3.129730 0.016421
H -1.559608 3.361248 1.553841
H 1.107719 5.299442 -1.402779
H 2.154074 5.113099 -0.003734
H -2.110604 5.599052 0.770441
H -1.579404 5.250773 -0.881600
H 0.110840 6.680492 0.353947
H 0.289604 5.409937 1.57191
2

D3-SP^{+}

E: -1404.78396686 a.u
C -0.932209 0.210548 -0.294180
C 1.439591 1.042849 -0.853319
C -1.657997 -0.955158 -0.352833
C -2.444527 -1.445287 -1.502254
C -1.960045 -1.870540 0.767528
C -3.051904 -2.684656 -1.127855
C -2.754060 -2.946979 0.256692
Fe -3.761432 -1.120854 0.075765
C -4.844051 -0.291617 1.689785
C -5.536277 -1.433453 1.147387
C -4.735769 0.690921 0.647020
C -5.836110 -1.158158 -0.233474
C -5.328699 0.153688 -0.547031
C 2.124203 -0.180664 -1.163440
C 1.841478 -1.534110 -0.717594
C 3.410211 -0.238682 -1.854565
C 2.862070 -2.409174 -1.227739
C 3.822622 -1.610046 -1.941323
Fe 3.745172 -1.014238 0.059072
C 5.682802 -1.340583 0.818961
C 4.737971 -2.130469 1.563364
C 5.333996 0.049147 0.991276
C 3.808623 -1.229102 2.196373
C 4.189320 0.110636 1.855186
C -0.242013 2.636065 -0.123536
C 0.956579 3.287956 -0.450740
C -1.499738 3.345357 0.311173
C 1.125180 4.786034 -0.341671
C -1.400740 4.876411 0.061168
C -0.019374 5.434709 0.478584
C -0.064637 1.188779 -0.354131
C 1.912780 2.329075 -0.933776
H -2.472123 -0.972226 -2.480843
H -1.566819 -1.770402 1.776535
H -3.672495 -3.304026 -1.772390
H -3.110162 -3.798384 0.833273
H -4.475664 -0.190189 2.708410
H -5.771557 -2.351480 1.681993
H -4.242360 1.656496 0.733950
H -6.337373 -1.831822 -0.925352
H -5.391699 0.651089 -1.512552
H 0.989621 -1.840215 -0.117498
H 3.912005 0.624703 -2.284027
H 2.909995 -3.485715 -1.076741
H 4.722634 -1.974641 -2.432370
H 6.508920 -1.722615 0.222657
H 4.720683 -3.216457 1.628492
H 5.845720 0.898935 0.544728
H 2.964280 -1.515767 2.819922
H 3.656796 1.016162 2.139641
H -2.377772 2.925461 -0.210190
H -1.667986 3.148778 1.389429
H 1.156282 5.211578 -1.363192
H 2.109927 5.016110 0.100149
H -2.206554 5.390063 0.608757
H -1.566707 5.077807 -1.011883
H 0.005109 6.528059 0.349274
H 0.144409 5.240114 1.553868

9^{+}

E: -230.50009073 a.u.
C -0.708023 1.072775 0.000002
C 0.708057 1.072840 0.000038
C 1.506110 -0.064327 0.000095
C -1.506059 -0.064361 -0.000007
C 2.272379 -1.034818 -0.000287
C -2.272378 -1.034785 0.000011
H -1.212675 2.047334 -0.000192
H 1.212569 2.047459 0.000124
H 2.932446 -1.889541 0.000921
H -2.932855 -1.889189 0.000031

TS-9-B^{+}

E: -230.45409636 a.u.
C -0.361998 0.598946 -1.276650
C 0.361998 -0.598946 -1.276650
C 0.701793 -1.070759 0.003469
C -0.701793 1.070759 0.003469
C 0.701793 -0.896013 1.256406
C -0.701793 0.896013 1.256406
H -0.636229 1.117584 -2.198768
H 0.636229 -1.117584 -2.198768
H 0.907104 -1.100938 2.299423
H -0.907104 1.100938 2.299423

D9-B+

E: -230.49295246 a.u.
C -0.754183 -2.000001 -0.008846
C 0.717655 -1.947245 -0.008059
C 1.195383 -0.680245 -0.007817
C 0.627764 0.548992 -0.008023
C -0.844370 0.495974 -0.008533
C -1.321971 -0.770913 -0.008949
H -1.239629 -2.979892 -0.009142
H 1.271795 -2.889939 -0.007846
H 1.112354 1.529249 -0.007778
H -1.398528 1.438638 -0.00859

TS-9-SP^{+}

E: -230.44213240 a.u.
C -0.569940 -1.151912 0.000076
C -1.596682 -0.239502 -0.000662
C -0.956105 1.045589 -0.000056
C 0.714388 -0.469846 0.001397
C 0.299916 1.354109 0.000657
C 1.978219 -0.415305 -0.001064
H -0.643365 -2.241502 0.001461
H -2.667820 -0.436171 -0.001598
H 1.054585 2.130109 -0.000827
H 3.037826 -0.191238 -0.001120

D9-SP+

E: -230.44238750 a.u.
C -3.261892 1.347907 -0.000522
C -1.981954 1.598874 -0.000689
C -1.348688 2.890072 -0.001554
C -2.399038 3.775834 -0.002125
C -3.662394 3.021013 -0.001629
C -4.935304 3.123439 -0.001813
H -3.991731 0.547405 0.000025
H -0.281526 3.106737 -0.001702
H -2.361214 4.866609 -0.002851
H -6.011736 2.992430 -0.002537

8^{+}

E: -1328.75685411 a.u.
C 0.0493230 3.6654900 0.1131720
C -0.1507880 5.0242000 0.4794200
C 0.9269390 5.9106080 0.5684730
C 2.2439650 5.4715690 0.2955970
C 2.4779350 4.1425280 -0.0600550
C 1.4013010 3.2133150 -0.1527430
H -1.1671300 5.3657630 0.6821250
H 0.7469890 6.9513630 0.8468580
H 3.0791690 6.1716140 0.3635120
H 3.4905030 3.7936380 -0.2709060
C -1.0611350 2.7943050 0.0017310
C 1.6699380 1.8686790 -0.4900820
C 1.9562260 0.7008890 -0.7707990
C 2.2695030 -0.6161850 -1.1441370
C 1.3710430 -1.7615780 -1.1273660
C 3.5904850 -1.1271870 -1.4837030
C 2.1041570 -2.9194890 -1.5546690
H 0.3155520 -1.7211270 -0.8685910
C 3.4727950 -2.5280050 -1.7750090
H 4.4935600 -0.5244040 -1.5489930
H 1.6968820 -3.9212700 -1.6758770
H 4.2834350 -3.1806370 -2.0930080
Fe 2.9480150 -2.2093690 0.2303060
C 4.2337360 -3.4139070 1.3944980
C 2.8638350 -3.7983390 1.6210420
C 4.3842670 -2.0438150 1.8181170
H 5.0164270 -4.0435330 0.9761520
H 2.4258010 -4.7705440 1.4042210
C 2.1735990 -2.6643600 2.1837310
C 3.1174300 -1.5932220 2.3165650
H 5.2978260 -1.4546780 1.7660970
H 1.1205910 -2.6262300 2.4557960
H 2.8936740 -0.5882730 2.6707580
C -2.0709080 2.0954760 -0.0975510
C -3.2354960 1.2901430 -0.2144750
C -3.1786560 0.0279390 -0.8758370
C -4.4778670 1.7371730 0.3245770
C -4.3229520 -0.7648740 -0.9892780
H -2.2326920 -0.3055140 -1.3068230
C -5.6233620 0.9438660 0.2139870
H -4.5260370 2.7042570 0.8276990
C -5.5279860 -0.2963110 -0.4389260
H -4.3101430 -1.7311970 -1.4927040
H -6.5839450 1.2614630 0.6188950
N -6.7484500 -1.1491700 -0.5530180
O -7.8083230 -0.6972820 -0.0740440
O -6.6199780 -2.2560970 -1.1175980

8^{+}(II)

E: -1328.75271536 a.u.
C 0.0898890 3.7451190 0.0454920
C -0.1211290 5.0905270 0.4567750
C 0.9320760 6.0089710 0.4741740
C 2.2293340 5.6144730 0.0766070
C 2.4694230 4.2994750 -0.3358090
C 1.4235600 3.3392680 -0.3421710
H -1.1264590 5.3924280 0.7551740
H 0.7480610 7.0372100 0.7928780
H 3.0490120 6.3359340 0.0903820
H 3.4694020 3.9924340 -0.6479550
C -0.9966820 2.8378680 0.0087850
C 1.6818030 2.0116560 -0.7771620
C 1.9465500 0.8754000 -1.1735140
C 2.2017900 -0.4270740 -1.6554420
C 2.1524890 -1.6610900 -0.8915340
C 2.7097450 -0.7711620 -2.9761460
C 2.5164720 -2.7471670 -1.7557370
H 1.8612620 -1.7345180 0.1542310
C 2.8586010 -2.2004670 -3.0438910
H 2.8851640 -0.0602350 -3.7805560
H 2.5478770 -3.7988830 -1.4781460
H 3.1797410 -2.7655070 -3.9167430
Fe 4.1637640 -1.4434780 -1.5991100
C 6.1110120 -1.9897970 -2.1709360
C 5.8140520 -2.5822960 -0.8914130

C 6.0476310 -0.5558320 -2.0187380
H 6.3428670 -2.5271450 -3.0883490
H 5.7685110 -3.6474480 -0.6730830
C 5.5727350 -1.5172820 0.0452870
C 5.7250820 -0.2724860 -0.6474930
H 6.2185870 0.1798630 -2.8021280
H 5.2961580 -1.6365070 1.0912280
H 5.5648340 0.7158210 -0.2203170
C -1.9793310 2.0945330 -0.0245430
C -3.1091430 1.2366140 -0.0663230
C -3.0005960 -0.0735290 -0.6208870
C -4.3684450 1.6763580 0.4408240
C -4.1126200 -0.9167270 -0.6660850
H -2.0387930 -0.4079770 -1.0135220
C -5.4815550 0.8330100 0.3963500
H -4.4544450 2.6783670 0.8642040
C -5.3367400 -0.4509710 -0.1559850
H -4.0598500 -1.9213550 -1.0846560
H -6.4543630 1.1438340 0.7764760
N -6.5244150 -1.3550610 -0.2032630
O -7.5988010 -0.9112990 0.2506140
O -6.3569420 -2.4917680 -0.6933600
